# Association Between Public Knowledge About COVID-19, Trust in Information Sources, and Adherence to Social Distancing: Cross-Sectional Survey

**DOI:** 10.2196/22060

**Published:** 2020-09-15

**Authors:** Ilona Fridman, Nicole Lucas, Debra Henke, Christina K Zigler

**Affiliations:** 1 Margolis Center for Health Policy Duke University Durham, NC United States; 2 Center for Health Measurement Department of Population Health Sciences Duke University School of Medicine Durham, NC United States

**Keywords:** health communication, COVID-19, trust in information sources, social distancing, behavior, coronavirus

## Abstract

**Background:**

The success of behavioral interventions and policies designed to reduce the impact of the COVID-19 pandemic depends on how well individuals are informed about both the consequences of infection and the steps that should be taken to reduce the impact of the disease.

**Objective:**

The aim of this study was to investigate associations between public knowledge about COVID-19, adherence to social distancing, and public trust in government information sources (eg, the US Centers for Disease Control and Prevention), private sources (eg, FOX and CNN), and social networks (eg, Facebook and Twitter) to inform future policies related to critical information distribution.

**Methods:**

We conducted a cross-sectional survey (N=1243) between April 10 and 14, 2020. Data collection was stratified by US region and other demographics to ensure representativeness of the sample.

**Results:**

Government information sources were the most trusted among the public. However, we observed trends in the data that suggested variations in trust by age and gender. White and older populations generally expressed higher trust in government sources, while non-White and younger populations expressed higher trust in private sources (eg, CNN) and social networks (eg, Twitter). Trust in government sources was positively associated with accurate knowledge about COVID-19 and adherence to social distancing. However, trust in private sources (eg, FOX and CNN) was negatively associated with knowledge about COVID-19. Similarly, trust in social networks (eg, Facebook and Twitter) was negatively associated with both knowledge and adherence to social distancing.

**Conclusions:**

During pandemics such as the COVID-19 outbreak, policy makers should carefully consider the quality of information disseminated through private sources and social networks. Furthermore, when disseminating urgent health information, a variety of information sources should be used to ensure that diverse populations have timely access to critical knowledge.

## Introduction

An unusual virus outbreak was documented in Wuhan, China in December 2019 [1]. By mid-March 2020, the World Health Organization (WHO) declared the COVID-19 outbreak to be a worldwide pandemic [1]. In early April, the number of COVID-19 cases in the United States exceeded 500,000 [2], and the death toll was approaching 30,000 [3]. In response, various states decided to implement serious measures to attempt to slow viral transmission. The US Centers for Disease Prevention and Control (CDC) asked individuals to wear masks, sanitize surfaces, and, most importantly, limit their social lives, including reducing face-to-face contacts and staying at least 6 feet apart from others [4]. Official stay-at-home orders were issued in at least 42 states, 3 counties, and 10 cities in the United States [5]. Americans were instructed to work from home when possible and limit nonessential trips and social gatherings [6]. Public places, including bars, restaurants, and playgrounds, were closed, and public events such as concerts and sports tournaments were canceled. The purpose of these restrictions was to save lives and avoid overburdening the health care system [7]. Evidence from data and predictive modeling showed that timely restriction of movements within countries with developed economies prevented more than 500,000 deaths [8]. Public adherence to restrictions can influence the success of the implementation of restrictive rules. Adherence depends on how well-informed people are about both the consequences of infection [9,10] and the steps that should be taken to prevent virus spread [11].

Previous research has shown that trust in sources is an essential component associated with both individual understanding of information and willingness to act on it [12]. Additionally, research in China has shown that people vary in their risk perception of COVID-19 depending on whether they received information from mass media or social media [13]. Therefore, in our work, we aimed to provide an overview of the sources people trusted early in the pandemic to inform policy makers on how to best disseminate critical information to reach different populations. We also explored the association between trust in different sources and accurate knowledge about COVID-19 to determine which information sources potentially need to improve the quality of their information to ensure that the public is well-informed about pandemic policies. Finally, following previous research that showed the association between understanding of COVID-19 and adherence to recommended risk-reducing behavior [14], we explored whether knowledge and trust were associated with adherence to social distancing behavior.

## Methods

### Data Collection

Data were collected via a cross-sectional national survey. An independent company that specializes in national data collection, Qualtrics Panels, implemented the recruitment procedures [15,16]. Individuals received an email invitation to the study if they preregistered for Qualtrics Panels and completed a baseline survey. Participants were informed about confidentiality, risks, and benefits at the beginning of the survey. Participants were then directed to the questionnaire; upon completion of the questionnaire, they received compensation. Qualtrics rewards participants with company points that can be redeemed for game rewards, gift cards, charitable contributions, or airline miles. Duke University’s institutional review board approved the study and deemed it exempt. The study design and analysis plan were preregistered at Open Science Framework [17].

To ensure representativeness of the sample, we stratified data collection by age, gender, and the following US regions: New England, Mid-Atlantic, East North Central, West North Central, South Atlantic, East South Central, West South Central, Mountain, and Pacific. The survey opened on April 10, 2020, and the data quality was evaluated after 200 responses. After this initial step, additional screening logic was implemented to exclude individuals aged <18 years. Likewise, participants who spent less than 6 minutes completing the survey were excluded from the survey. Between April 13 and April 14, 2020, 1000 participants completed the survey. Data collected on April 10 and April 13 to 14 were included in the analysis.

### Survey

The survey was part of a larger study to explore how Americans were responding to CDC recommendations and guidelines during the pandemic. Participants reported their demographics, current work/income circumstances, location, and health status, including conditions that were associated with increased risks of dying from COVID-19. In the current work, we focused on exploring the association between trust in information sources, knowledge about COVID-19, and adherence to social distancing. A full copy of the survey can be found in Open Science Framework (OSF) Registries [17].

#### Trust in Information Sources About COVID-19

To evaluate trust in different information sources, we provided examples of government-affiliated sources, privately affiliated sources, and social networks. We asked participants to rank their trust on a 5-point Likert scale that ranged from 1 (“not trustworthy at all”) to 5 (“extremely trustworthy”). Additionally, an “I don’t know” option was available for participants who were unfamiliar with the provided examples. For government-affiliated sources, we chose the following examples: The White House, the CDC, the US Food and Drug Administration (FDA), the WHO, and local health departments. To evaluate participant trust in privately affiliated media, we used the MarketWatch summary [18], which sorts sources into two dimensions: political orientations and facts vs opinions. The examples represented liberal, conservative, and neutral sides. In each political domain, two sources were included: a source that was classified as providing facts and a source that was classified as providing opinions. The liberal sources were the *New York Times* (facts) and MSNBC (opinions); the conservative sources were a news website, The Hill (facts), and Fox News (opinions); and the neutral sources were Reuters (facts) and CNN (opinions). Examples of social networks include Facebook and Twitter.

To ensure the inclusiveness of the news sources, we allowed participants to indicate other sources that they trusted the most via open-response items. Participants were instructed to specify if a trusted source was not listed in a survey section (eg, social networks) and then provide the name of their trusted source (eg, “Reddit”) and rate the source on the same scale as the other sources. For analysis, we considered a source as “trusted” when participants rated it as “trustworthy” or “extremely trustworthy.”

#### Frequency of Accessing Information About COVID-19

To evaluate whether participants followed news about COVID-19, we asked them to rate their agreement with the following statement: “I follow updates about the coronavirus and the outbreak closely.” This item was scored on a 5-point scale that ranged from “strongly disagree” to “strongly agree.” Participants also reported how frequently they checked the news on an 8-point scale ranging from “never” to “5 or more times a day.” Furthermore, we provided examples of information sources (discussed above) and asked the participants to rate how frequently they checked each source of information in the past week. Participants reported the frequency on a 5-point scale ranging from “never” to “multiple times a day.”

#### Knowledge About COVID-19

To evaluate the participants’ knowledge about COVID-19, seven items were adopted from previous research on COVID-19 by RTI International [19,20]. Five additional items were designed based on current CDC guidelines and common myths about COVID-19 that circulated in the media at the end of March 2020. The response mode of the scale included binary endpoints of “true” and “false.” The scale consisted of items related to facts and myths about the virus, such as “Antibiotics can be used to treat the coronavirus” and “Most people who are infected with the coronavirus die from it.” The scale also included items related to risk-reducing behavior, such as “I cannot be infected if I wear a mask” and “By limiting the contact I have with people outside my household, I could prevent somebody's death.” The knowledge score was calculated using the percent of correct responses to all 12 items (listed in Multimedia Appendix 1).

#### Social Distancing

We asked participants about the frequency of seven specific social distancing behaviors recommended by the CDC at the beginning of April to prevent the spread of COVID-19 [4]. Participants reported how often they engaged in specific behaviors over the past seven days on a 5-point scale ranging from “not at all” to “several times a day.” Individual negative behaviors included “Hugging or touching people who do not live with me,” “Standing or walking close (within arm’s length) to someone who does not live with me,” “Meeting face-to-face with people who do not live with me,” “Going to gatherings with five or more people,” “Going inside someone else’s house,” and “Having friends or family over to visit” (see Multimedia Appendix 1). If participants reported leaving their house at least once in the past week, they were also asked how often they stayed six feet away from people who did not live in their household. For analysis, participants were considered to be adherent to all social distancing behaviors if they responded “not at all” to all negative behavior questions and “always” stayed six feet away from people outside their household (or did not leave their house in the past week).

### Data Analysis

Demographics, location, work, and health status were reported as both frequencies and percentages.

#### Trust in Information Sources About COVID-19

For each example of an information source, we summarized the percentage of people who trusted the source. Further, the percentages of participants who trusted (versus those who did not trust) each source were determined for age, race, and region groups. For age groups, we were specifically interested in older Americans (>65 years of age), as the CDC considers them to be a vulnerable population.

#### Frequency of Accessing Information About COVID-19

We reported the percentage of participants who reported “closely” following the news about COVID-19 and the percentage of participants who checked the news about COVID-19 at least “once a day.” We also summarized how often individuals reported checking specific information sources.

#### Knowledge About COVID-19

We presented the total percentages of correct responses to all knowledge items and correct answers by item. The Spearman correlation was used to estimate the association between correct responses (“accurate knowledge”) and trust in different information sources.

#### Social Distancing

The percentage of people who adhered to social distancing behavior (as defined by the CDC) was reported, along with the frequencies of adherence to each specific behavior. Chi-square statistics and significance levels were used to evaluate whether there were more adherent participants among those who trusted a particular information source than among those who did not trust a particular source.

#### Elastic Net Regression

We used *trust* in information sources to model *accurate* knowledge about COVID-19. The primary goal was not prediction per se; rather, we aimed to identify the information sources that contributed the most to the accuracy of participant knowledge about COVID-19 when all the sources were simultaneously included in the prediction model. Participant trust in each information source were the independent variables, while the percentage of correct responses to COVID-19 knowledge items was the dependent variable. We chose elastic net regression because it allowed us to determine the model that fits our multiparameter data and highlighted the most influential information sources that predicted participant knowledge about COVID-19 [21,22]. This approach uses regularization parameters for shrinking the influence of “weak” information sources to “0,” leaving only information sources that had a “strong” association with knowledge in the model. Additionally, this approach controls for potential multi-collinearity by considering correlations amongst the independent variables.

Data were randomly split into training and test data sets (80% and 20%, respectively). In the model, we used ordinary least squares regression. To evaluate the model fit, we used *R*^2^ and root mean square error (RMSE) to tune these parameters for a better fit between the model and the data.

The same approach was utilized to establish the information sources that contributed most strongly to adherence to social distancing behaviors. In this case, the elastic net regression used logistic regression with regularization parameters. To evaluate the model fit, we used the area under the curve (AUC), which illustrates how well a model can predict the dependent variable (here, adherence to social distancing). We tuned the regularization parameters to maximize the AUC. For both models, elastic net regression was implemented in the glmnet package in R [23].

#### Exploratory Analysis

**Figure 1:**
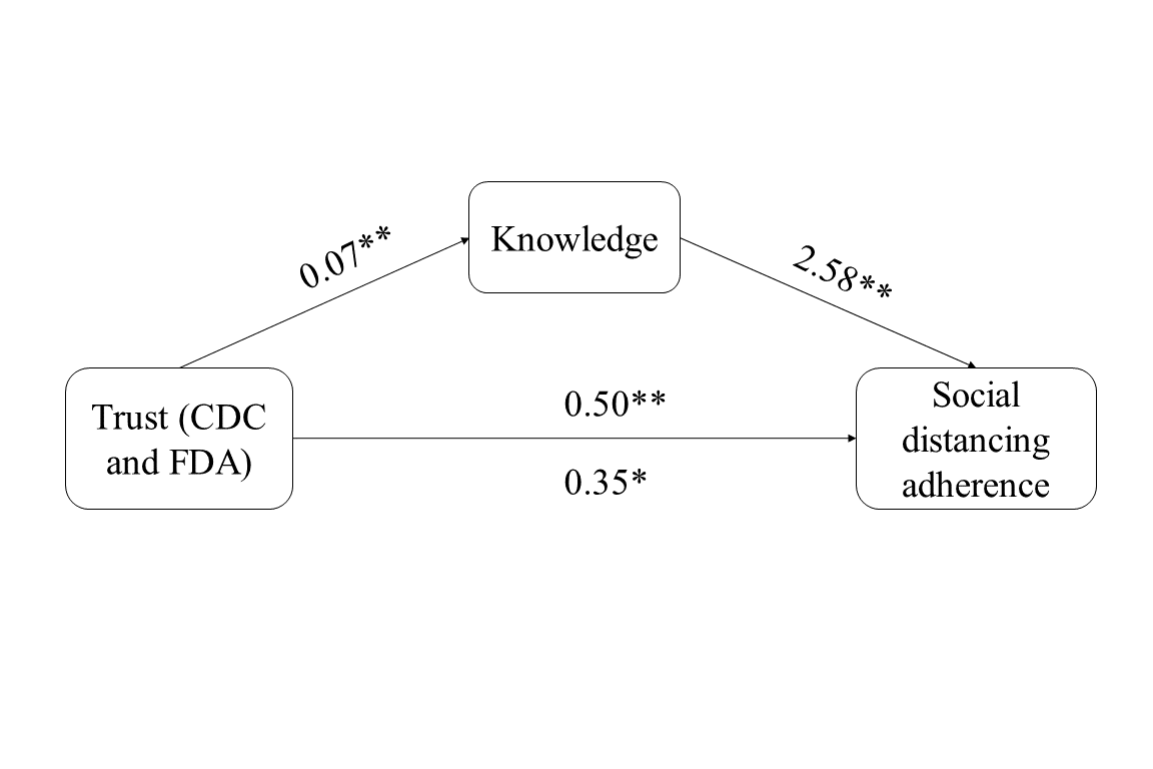
Indirect effects of trust in CDC and FDA information sources and adherence to social distancing. CDC: US Centers for Disease Control and Prevention; FDA: US Food and Drug Administration. *Significant at .05, **significant at .001.

Although not preregistered, we also explored whether knowledge mediated the relationship between trust in an information source and adherence to social distancing. The source that contributed to the accuracy of knowledge the most, as defined by elastic net regression coefficients, was used as a predictor in our mediation analysis. We fit three models to the data with adherence to social distancing as an outcome and knowledge as a mediator (see Figure 1). Model 1 included a binary logistic regression of trust (X) on participant adherence to social distancing (Y), Model 2 was a linear regression of trust (X) on participant knowledge about COVID-19 (M), and Model 3 was a binary logistic regression predicting adherence (Y) by trust (X) and knowledge (M). An indirect effect and bootstrap procedure were conducted using the “process” macro [24].

## Results

The total sample included 1243 participants. Main parameters such as age, gender, race, location, and income were closely aligned with the general US population as per the 2018 Census [25] and are reported in Table 1.

**Table 1:** Demographic and descriptive statistics of the study participants (N=1243).

Characteristic		Value
**Age (years)**		
	20-40, n (%)	579 (48.3)
	40-60, n (%)	353 (29.4)
	60-80, n (%)	267 (22.3)
	Mean (SD)	44 (16)
**Gender, n (%)**		
	Female	648 (52.1)
	Male	580 (46.7)
	Other	15 (1.2)
**Race/ethnicity^a^, n (%)**		
	White	888 (72.0)
	Black or African American	162 (13.1)
	Asian	85 (6.9)
	Hispanic or Latino	92 (7.5)
	American Indian and Alaska Native	35 (2.8)
	Native Hawaiian and Other Pacific Islander	7 (<1.0)
**Income (US $), n (%)**		
	Less than 14,999	192 (15.4)
	15,000-74,999	715 (57.6)
	75,000 to 99,999	143 (11.5)
	100,000 to 149,999	110 (8.9)
	More than 150,000	81 (6.6)
	Did not answer	2 (0.1)
**Location, n (%)**		
	New England	57 (4.6)
	Mid-Atlantic	171 (13.8)
	East North Central	168 (13.5)
	West North Central	79 (6.4)
	South Atlantic	256 (17.1)
	East South Central	75 (6.0)
	West South Central	150 (12.1)
	Mountain	90 (7.3)
	Pacific	193 (15.6)
	Did not answer	4 (0.3)
**Under stay-at-home order, n (%)**		
	Yes	979 (78.8)
	No	192 (15.4)
	Not sure	72 (5.8)
**Employment status, n (%)**		
	Employed full-time	484 (38.9)
	Employed part-time	135 (10.9)
	Retired	190 (15.3)
	On disability	71 (5.7)
	Self-employed	95 (7.6)
	Unemployed	268 (21.6)
**Work status, n (%)**		
	Working from home	422 (33.9)
	Not working from home	292 (23.5)
	Essential worker	343 (27.6)
	Nonessential worker	346 (27.8)
**Chronic health condition or care provider, n (%)**		
	Has chronic health condition	497 (40.0)
	Lives with person with chronic health condition	455 (36.6)
	Taking care of person outside household	158 (12.7)
**Infected/suspected infected with COVID-19, n (%)**		
	Yes	53 (4.3)
	No	1106 (89.0)
	Maybe	84 (6.8)

^a^Data do not sum to 1243 because more than one option could be selected.

### Trust in Information Sources About COVID-19

We found that the majority of participants trusted government sources (Table 2). Less than one-third of the participants trusted social media with regard to information about COVID-19. Older adults were more likely to trust government sources compared to younger adults. Conversely, middle-aged and younger populations trusted private sources and social networks more than older populations. On average, individuals who identified as White reported more trust in government sources than non-White participants, who trusted more private sources and social networks.

**Figure 2:**
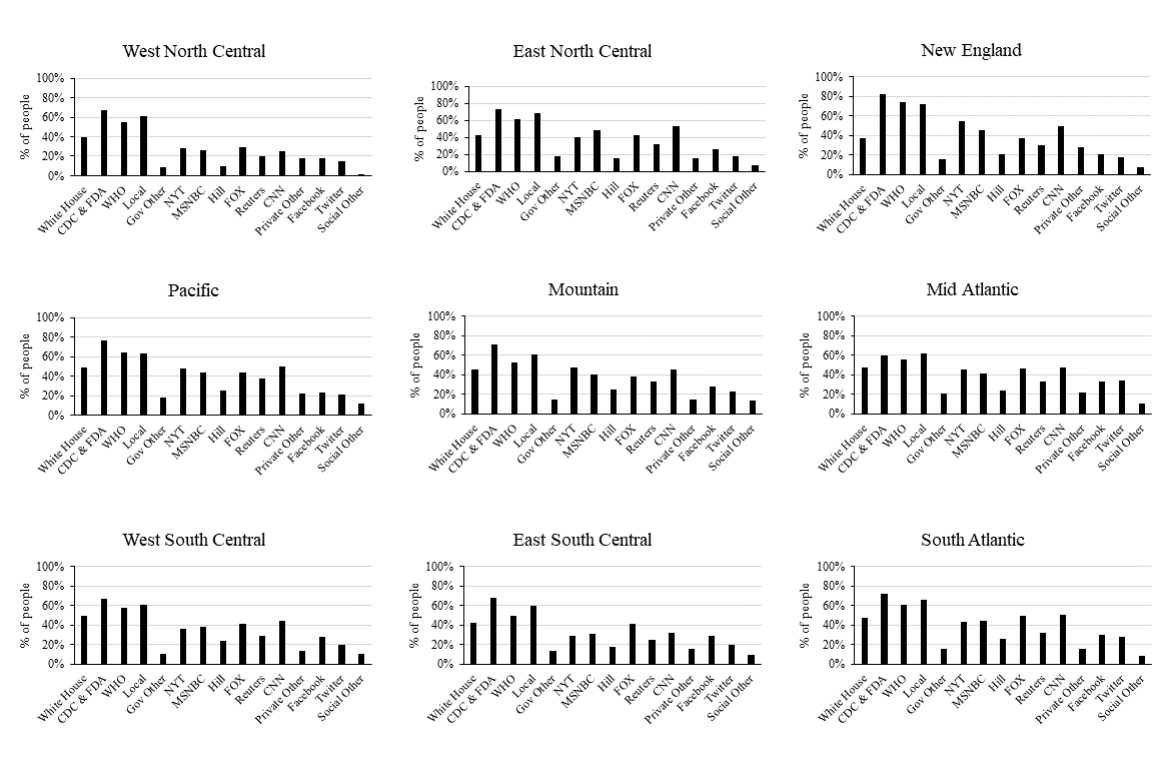
Percentages of participants who trusted in information sources (out of all people in a given region) presented by region and by information source. CDC: US Centers for Disease Control and Prevention; FDA: US Food and Drug Administration; Gov: government; NYT: New York Times; WHO: World Health Organization.

The trends of trust in the information sources were similar between regions. Notably, the highest prevalence rate of COVID-19 at the time of data collection was in the Mid-Atlantic region. However, this region had the lowest percentage of people (n = 102 out of 171; 60.0%) who trusted CDC and FDA sources compared to the average population (n=873, 70.3%); see Figure 2.

**Table 2:** Numbers of participants who trust each information source (N=1243), n (%). Trust was defined as binary (trust vs no trust) regarding providing accurate information about COVID-19.

Domain and information sources		Trust by total sample^a^		Trust by age group^b^ (years)						Trust by race		
					<25	25-40	41-50	51-64	≥65		White	Non-White
**Government sources**												
	CDC^c^ and FDA^d^		874 (70.3)		99 (64.7)	298 (64.8)	*142 (75.1)*	*222 (79.0)*	*111 (75.5)*		*645 (72.2)*	229 (65.6)
	Local health department		792 (63.7)		81 (52.9)	280 (60.0)	*129 (68.3)*	*195 (69.4)*	*105 (71.4)*		*574 (64.2)*	218 (62.5)
	WHO^f^		736 (59.2)		*93 (60.8)*	269 (57.6)	*113 (59.8)*	*177 (63.0)*	*82 (55.8)*		529 (59.2)	207 (59.3)
	White House		569 (45.8)		65 (42.5)	205 (43.9)	*94 (49.7)*	*134 (47.7)*	*69 (46.9)*		*426 (47.7)*	143 (41.0)
	Other		196(15.8)		18 (11.8)	*91 (19.5)*	*32 (16.9)*	36 (12.8)	18 (12.2)		131 (14.7)	*65 (18.6)*
**Private sources**												
	CNN		577 (46.4)		64 (42.8)	*229 (49.0)*	*88 (46.6)*	*133 (47.3)*	60 (40.8)		389 (43.5)	*188 (53.9)*
	FOX		534 (42.9)		63 (41.1)	*201 (43.0)*	*90 (47.6)*	*122 (43.4)*	57 (38.8)		*392 (43.9)*	142 (40.7)
	New York Times		523 (42.0)		*70 (45.8)*	*208 (44.5)*	*81 (42.9)*	105 (37.4)	55 (37.4)		352 (39.4)	*171 (49.0)*
	MSNBC		515 (41.4)		56 (36.7)	*204 (43.7)*	*89 (47.1)*	108 (38.4)	55 (37.4)		346 (38.7)	*169 (48.4)*
	Reuters		391 (31.5)		37 (24.2)	*151 (32.3)*	*64 (33.9)*	*89 (31.7)*	49 (33.3)		272 (30.4)	*119 (34.1)*
	The Hill		273 (22.0)		*37 (24.2)*	*129 (27.6)*	*44 (23.3)*	43 (15.3)	19 (12.9)		179 (20.0)	*94 (26.9)*
	Other		221 (17.8)		20 (13.0)	*96 (20.6)*	*35 (18.5)*	49 (17.4)	20 (13.6)		158 (17.7)	*63 (18.1)*
**Social networks**												
	Facebook		335 (27.0)		335 (27.0)	38 (24.8)	161 (34.5)	62 (32.8)	60 (21.4)		226 (25.3)	*109 (31.2)*
	Twitter		290 (23.3)		290 (23.3)	44 (28.8)	140 (30.0)	51 (27.0)	39 (13.9)		189 (21.1)	*101 (28.9)*
	Other		115 (9.3)		115 (9.3)	14 (9.2)	58 (12.7)	24 (12.7)	14 (5.0)		73 (8.2)	*42 (12.0)*

^a^Percentages were calculated as the ratio of people who rated the source as trusted to the total sample size.

^b^Percentages for age and race were calculated as the ratio of people who rated the source as trusted to the sample size of each subgroup.

^c^CDC: US Centers for Disease Control and Prevention.

^d^FDA: US Food and Drug Administration.

^e^Italics highlight the subgroups in which the percentages of people who trusted the source were equal to or greater than that of the total sample.

^f^WHO: World Health Organization.

### Frequency of Accessing Information About COVID-19

The majority of participants reported following the news about the COVID-19 outbreak “closely” (n=998, 80.3 % strongly agree/agree) and checking updates about the COVID-19 outbreak at least once a day (n = 1054, 84.6%). Table 3 presents the frequencies of access to different sources.

**Table 3:** Frequency at which participants reported checking information sources to obtain information about COVID-19 by source of information (N=1243), n (%).

Domain and name of the source		Never	Once a week	Several times a week	Daily	Multiple times a day
**Government sources**						
	White House press briefings	342 (27.5)	226 (18.2)	268 (21.6)	321 (25.8)	86 (6.9)
	Federal health agencies (CDC^a^ and FDA^b^)	226 (18.2)	252 (20.3)	341 (27.4)	315 (25.3)	109 (8.8)
	International organization (WHO^c^)	389 (31.3)	259 (20.8)	257 (20.7)	236 (19.0)	102 (8.2)
	State/local health agencies	307 (24.7)	215 (17.3)	285 (22.9)	330 (26.6)	106 (8.5)
**Private sources**						
	FOX News or The Hill	406 (32.7)	173 (13.9)	240 (19.3)	265 (21.3)	159 (12.8)
	MSNBC or the *New York Times*	491 (39.5)	168 (13.5)	230 (18.5)	236 (19.0)	118 (9.5)
	Reuters or CBS News	418 (33.6)	168 (13.5)	241 (19.4)	302 (24.3)	114 (9.2)
	Community/local news	162 (13.0)	180 (14.5)	268 (21.6)	466 (37.5)	167 (13.4)
**Social networks**						
	Facebook	465 (37.4)	133 (10.7)	167 (13.4)	307 (24.7)	171 (13.8)
	Twitter	750 (60.3)	92 (7.4)	135 (10.9)	163 (13.1)	103 (8.3)
	Podcasts	845 (68.0)	95 (7.6)	128 (10.3)	117 (9.4)	58 (4.7)
	Blogs	855 (68.8)	91 (7.3)	112 (9.0)	118 (9.5)	67 (5.4)
	Family	284 (22.8)	223 (17.9)	337 (27.1)	275 (22.1)	124 (10.0)

^a^CDC: US Centers for Disease Control and Prevention.

^b^FDA: US Food and Drug Administration.

^c^WHO: World Health Organization.

### Knowledge About COVID-19

The mean COVID-19 knowledge score was 85% (SD 17%); this indicates that on average, people responded to 10 out of 12 questions correctly. However, only 306/1243 participants (30.6%) answered all the knowledge questions correctly. Some items were more difficult than others, as represented by the lower percentages of people who answered them correctly (Table 4).

**Table 4:** Numbers of participants who correctly answered individual items on the scale measuring knowledge about COVID-19 (N=1243), n (%). F: correct answer is false; T: correct answer is true.

Item	Correct responses
The United States is weeks away from having an FDA approved vaccine for coronavirus (F)	753 (60.6)
Antibiotics can be used to treat the coronavirus (F)	878 (70.6)
Most people who are infected with the coronavirus die from it (F)	991 (79.7)
I cannot be infected if I wear a mask (F)	1048 (84.3)
People do not transmit the virus if they don’t have symptoms (F)	1053 (84.7)
Eating garlic can lower your chances of getting infected with the coronavirus (F)	1054 (84.8)
Most people who are infected with the coronavirus recover from it (T)	1070 (86.1)
By limiting the contact I have with people outside my household, I could prevent somebody's death (T)	1128 (90.8)
The main symptoms of the coronavirus are fever and cough (T)	1142 (91.9)
People of all ages can be infected with the coronavirus (T)	1155 (92.9)
People of all racial and ethnic groups can become infected with the coronavirus (T)	1163 (93.6)
To protect myself I need to wash hands frequently (T)	1173 (94.4)

Using correlations, we found a positive association between knowledge and trust in government sources such as the CDC, the FDA, local health departments, and the WHO (Table 5). There was a negative association between accurate knowledge about COVID-19 and participants’ trust in private information sources and social media.

**Table 5:** Associations of trust in individual information sources with knowledge about COVID-19 and with adherence to social distancing (N=1243).

Domain and name of the source		Total participants who trusted the source, n (%)	Knowledge about COVID-19					Social distancing			
			Trusted source and answered all 12 knowledge questions correctly, n (%)		Did not trust source and answered all 12 knowledge questions correctly, n (%)	Spearman correlation of knowledge and trust, ρ	*P* value	Adhered to social distancing and trusted source, n (%)	Adhered to social distancing but did not trust source, n (%)	Adherence and trust, chi-square (1242)	*P* value
**Government sources**											
	*CDC* ^a^ *and FDA* ^b^	*874 (70.3)* ^c^		*298 (34.1)*	*82 (22.2)*	*0.18* ^d^	*0.000*	*306 (35.0)*	*91 (24.7)*	*12.77* ^d^	*0.000*
	Local health department	792 (63.7)		260 (32.8)	120 (26.6.)	0.10^e^	0.000	275 (34.7)	122 (27.1)	7.78^d^	0.005
	WHO^f^	736 (59.2)		242 (32.9)	138 (27.2)	0.08^d^	0.007	255 (34.6)	142 (28.0)	6.09^e^	0.014
	White House	569 (45.8)		143 (25.1)	237 (35.1)	–0.12^d^	0.000	178 (31.3)	219 (32.5)	0.21	0.649
	Other	196(15.8)		33 (16.8)	347 (33.1)	–0.19^d^	0.000	46 (23.5)	351 (33.5)	7.68^d^	0.006
	None	180 (15)		37 (20.6)	343 (32.3)	–0.14^c^	0.000	46 (25.6)	351 (33.0)	3.95^e^	0.047
**Private sources**											
	CNN	577 (46.4)		178 (30.8)	202 (30.3)	–0.04	0.124	182 (31.5)	215 (32.3)	0.08	0.780
	FOX	534 (42.9)		124 (23.2)	256 (36.1)	–0.17^d^	0.000	163 (30.5)	234 (33.0)	0.86	0.353
	New York Times	523 (42.0)		171 (32.7)	209 (29.0)	–0.04	0.198	171 (32.7)	226 (31.4)	0.24	0.626
	MSNBC	515 (41.4)		164 (31.8)	216 (29.7)	–0.05	0.067	171 (33.2)	226 (31.0)	0.65	0.421
	Reuters	391 (31.5)		115 (29.4)	265 (31.1)	–0.12^d^	0.000	109 (27.9)	288 (33.8)	4.33^e^	0.037
	The Hill	273 (22.0)		51 (18.7)	329 (33.9)	–0.27^d^	0.000	68 (24.9)	329 (33.9)	7.95^d^	0.005
	Other	221 (17.8)		51 (23.1)	329 (32.2)	–0.13^d^	0.000	64 (29.0)	333 (32.6)	1.10	0.295
	None	293 (24)		76 (25.9)	304 (32.0)	–0.01	0.705	97 (33.1)	300 (31.6)	0.24	0.624
**Social networks**											
	Facebook	335 (27.0)		60 (17.9)	320 (35.2)	–0.29^d^	0.000	87 (26.0)	310 (34.1)	7.52^d^	0.006
	Twitter	290 (23.3)		46 (15.9)	334 (35.0)	–0.31^d^	0.000	69 (23.8)	328 (34.4)	11.55^d^	*0.001*
	Other	115 (9.3)		15 (13.0)	365 (32.4)	–0.21^d^	0.000	20 (17.4)	337 (33.4)	12.34^d^	*0.000*
	None	796 (64)		291 (36.6)	89 (19.9)	0.29^d^	0.000	280 (35.2)	117 (26.2)	10.53^d^	0.001

^a^CDC: US Centers for Disease Control and Prevention.

^b^FDA: US Food and Drug Administration.

^c^Italics illustrate sources that were suggested by elastic net regression to be associated with knowledge and adherence while controlling for trust in all other sources.

^d^Significant at .001.

^e^Significant at .05.

^f^WHO: World Health Organization.

Elastic net regression suggested that seven information sources had the strongest associations with participant knowledge. The standardized regression coefficients illustrated a positive association between knowledge and the CDC/FDA (β=.06), local health department (β=.01), and a negative association with “other” government sources (β=–.01), The Hill (β=–.07), Facebook (β=–.03), Twitter (β=–.06) and other social networks (β=–.02). The model included the following parameters (RMSE_training_=0.14, RMSE_test_ = 0.16, *R*^2^_training_=0.27, *R*^2^_test_=0.22).

### Social Distancing

 In total, only 32% of participants reported adhering to all seven recommended social distancing behaviors. The most compliant behavior was avoiding gatherings with 5 or more people. The least compliant behaviors were meeting people face-to-face and walking close to others. Table 6 shows the participants’ reported frequency of engaging in the six negative social distancing behaviors. For the positive social distancing behavior, staying 6 feet from other people, the 1243 participants reported frequencies of always (n=801, 64.4%), usually (n=287, 23.1%), sometimes (n=104, 8.4%), rarely (n=24, 1.9%), and never (n=27, 2.2%). The statistics includes these who did not leave the house in past seven days. Participants were considered adherent if they did not engage in risk-increasing behaviors or always stayed 6 feet apart from other people.

**Table 6:** Self-reported frequency of social distancing behavior not recommended by the US Centers for Disease Control and Prevention (N=1243), n (%). Note that the statistics include people who did not leave the house for seven days.

Behavior	Social distancing adherence scale				
	Not at all	Once a week	Several times a week	Daily	Several times a day
Went to a gathering with 5 or more people	919 (73.9)	110 (8.8)	83 (6.7)	71 (5.7)	60 (4.8)
Hugged or touched someone who does not live with you	909 (73.1)	103 (8.3)	88 (7.1)	97 (7.8)	46 (3.7)
Went inside someone else’s house	854 (68.7)	152 (12.2)	104 (8.4)	84 (6.8)	49 (3.9)
Had friends or family over to visit	841 (67.7)	159 (12.8)	101 (8.1)	88 (7.1)	54 (4.3)
Stood or walked close to someone who does not live with you	678 (54.5)	230 (18.5)	167 (13.4)	97 (7.8)	71 (5.7)
Met face-to-face with people who don’t live with you	673 (54.1)	227 (18.3)	171 (13.8)	114 (9.2)	58 (4.7)

The percentage of people who adhered to social distancing behaviors was *higher* among participants who trusted government sources such as the CDC and FDA, local health departments, and the WHO than among those who did not trust these sources (Table 5). In contrast, the percentage of people who adhered to social distancing behaviors was *lower* among participants who trusted some private sources and social networks than among those who did not trust these sources.

Elastic net regression suggested that four variables had the strongest association with participant adherence. Final standardized regression coefficients included positive associations with trust in the CDC and FDA (β=.02) and the local health department (β=.01), and negative associations with trust were observed for Twitter (β=–.02), and “other” social networks (β=–.05). However, the model had low explanatory power when predicting adherence (AUC_training_=63, AUC_test_=59). We suggested testing a mediation effect to evaluate whether trust in information sources is associated with adherence via increasing knowledge about COVID-19, as reported below.

### Exploratory Analysis

We observed that trust in the CDC and FDA was associated with more accurate knowledge about COVID-19 and adherence to all social distancing behaviors (see Table 7 and Figure 1, Model 1). We found that as knowledge increased, so did the participants’ likelihood of reporting that they tended to distance from those who did not live in their household. When knowledge was included in the regression model, the predicted relationship between trust and adherence decreased in size, indicating partial mediation (Model 3). The odds ratio (OR) for knowledge in our final model equaled 1.24, meaning that for every additional question answered correctly, we would expect a 24% increase in the odds of adhering to all recommended social distancing behaviors.

Subsequent exploratory analysis showed that health status, income, being under a stay-at-home order, and working from home were not associated with adherence, while age had a significant association with adherence to social distancing guidelines (β=.02; SE .004; *P*<.001). Including age in the mediation model did not change significance levels reported in the baseline model; the indirect effect remained significant (*b*=0.13*). The OR for the knowledge variable was 1.21.

**Table 7:** Results of the mediation analysis of trust in the CDC and FDA (X), knowledge about COVID-19 (M), and adherence to social distancing behaviors (Y). Null prediction for adherence to all social distancing behavior was 32%; reported coefficients are unstandardized.

Model		B	*P* value	SE	Bootstrap 95% CI	*R^2^* ^a^	Odds ratio
**Model 1: Adherence (yes/no; binary logistic regression)**						0.02	
	Constant	–1.12^b^	<.001	0.12	N/A^c^		0.33
	Trust in CDC^d^ and FDA^e^	0.50^b^	<.001	0.14	N/A		1.65
**Model 2: Knowledge (linear regression)**						0.03	
	Constant	0.80^b^	<.001	0.01	N/A		
	Trust in CDC and FDA	0.07^b^	<.001	0.01	N/A		
**Model 3: Adherence (yes/no: binary logistic regression)**						0.06	
	Constant	–3.23^b^	<.001	0.38	N/A		0.04
	Trust in CDC and FDA	0.35^f^	.02	0.14	N/A		1.42
	Knowledge	2.58^b^	<.001	0.43	N/A		1.24
	Indirect effect	0.18^b^	<.001	0.04	0.11-0.27		1.20

^a^For logistic regression models, *R*^2^ is the version proposed by Nagelkerke.

^b^Significant at .001.

^c^N/A: not applicable.

^d^CDC: US Centers for Disease Control and Prevention.

^e^FDA: US Food and Drug Administration.

^f^Significant at .05.

## Discussion

### Principal Findings

In a cross-sectional survey, we explored which information sources the public trusted with regard to health information and how the trust in specific sources was associated with accurate knowledge about COVID-19 and adherence to recommended social distancing behaviors. We found that the majority of participants trusted government information sources, such as the CDC, FDA, local health departments, and the WHO. Although concerns are increasing about the public’s use of social networks to learn about the risks of COVID-19 [26,27], we found that only 36% of people trusted information in social networks. Although not explicitly tested, general trends in our data suggested that trust in information sources varied by age and race. White and older respondents were more likely to trust government sources than non-White and younger respondents, who were more likely to trust private sources and social media. These findings highlight the importance of using different channels to distribute timely health information that reaches diverse populations.

Further, we investigated whether trust in specific information sources was associated with participant knowledge about COVID-19. Trust in government sources (the CDC, the FDA, and local health departments) had a positive association with accurate knowledge about COVID-19, whereas trust in private sources and social networks had a negative association. Consistent with our findings, other studies have shown that private media sources distribute messages that can reduce public trust in scientific knowledge and health policies [28,29]. Several studies have shown that social networks can become a platform for the distribution of misinformation. Kouzy and colleagues [30] manually evaluated tweets at the beginning of the pandemic and identified that 25% of tweets contained misinformation. In addition, another study showed an association between beliefs in conspiracy theories and social media use [31].

We also identified that adherence to social distancing guidelines was positively associated with trust in government information sources and further explored the mechanism behind this association via mediation analysis. We found that knowledge about COVID-19 partially mediated this relationship. Similar relationships between trust, knowledge, and adherence were found in a cross-sectional survey conducted in China [14]. The researchers conducted a path analysis using a structural model approach and found that trust in formal and informal sources increased participants’ awareness about SARS-CoV-2; then, in turn, the awareness was associated with social distancing measures. Noteworthily, trust and accurate knowledge explained only a fraction of the variability in adherence to social distancing. For instance, if participants answered 50% of the knowledge questions correctly, the model suggested only a 17% probability of adherence to social distancing behavior if the participants trusted the CDC and FDA. In the same vein, if the participants answered all the knowledge questions correctly, there was still only an approximately 44% probability of adherence to social distancing behaviors. It was surprising that our elastic net regression model, which included trust in all sources, had low predictive power, specifically in regard to predicting adherence to social distancing. However, the model served well for the main goal of the analysis by distilling the predictive value of the specific sources that contributed the most to knowledge and adherence. Further research should investigate other factors that influence adherence to social distancing, including social, logistic, economic, and political issues.

Our results support several practical recommendations that could help increase knowledge about COVID-19 and improve the adoption of risk-reducing behavior. First, our work showed that trust in information sources was associated with participants’ knowledge about COVID-19. Thus, maintaining and increasing trust in information sources is an important task for policy makers. During unprecedented events such as pandemics, health messages might change and, at times, contradict previously reported information and recommendations. For instance, early on in the pandemic, the US Surgeon General communicated that face masks were “NOT effective” [32]. However, the CDC later recommended wearing masks as a mandatory requirement for people who visited public places [33]. To maintain trust in information sources, policy makers should communicate information only when there is a strong scientific consensus. Building relationships with well-established, trusted scientific experts could help in achieving this goal [34]. Furthermore, it is important to acknowledge the uncertainty of delivered information. For instance, at the beginning of April 2020, Dr Anthony Fauci, head of the National Institute of Allergy and Infectious Diseases at the National Institutes of Health, said about asymptomatic cases: “It’s somewhere between 25 and 50 percent, and trust me, that is an estimate. I don’t have any scientific data yet” [35]. It is expected but not yet tested that communicating uncertainty will help individuals be more open about updating their beliefs when more information becomes available.

Second, we noticed that trust in specific sources of information varied among people by age and by ethnic and racial characteristics. Therefore, policy makers should consider communicating information through multiple sources. Establishing and maintaining relationships with journalists and private sources and maintaining organized and updated social media accounts could help ensure that individuals with diverse backgrounds receive critical health messages in a timely fashion. Policy makers could also consider novel approaches toward information distribution, such as crowdsourcing. For instance, YouTube encouraged its viewers to create video clips about activities they were engaging in while staying at home (eg, singing, meditating) and played them as a social advertisement to promote adherence to stay-at-home orders [36]. Although these campaigns are interesting, their effectiveness must be evaluated in future research.

Third, we found negative associations between participants’ knowledge and trust in private and social media sources. We believe that this finding supports and echoes other voices calling for improvement of the quality of the information disseminated through these sources. For instance, media platforms can flag unverified information and disrupt automated accounts (bots) that distribute false information [37]. Recently, Twitter added fact-checking links to individual tweets that provide unverified or suspicious information [38]. Additionally, individual users of social networks can receive “accuracy reminders” that encourage them to verify the trustworthiness of their sources. This approach has been shown to be effective in reducing participants’ intention to repost COVID-19–related misinformation [39].

The data collection occurred shortly after stay-at-home orders were implemented in the majority of US states, and Americans were constantly receiving updates on the changing policies related to COVID-19. Previous research has shown that the beginning stages of pandemics attract the most attention [40]. This is consistent with our study, as the majority of participants reported checking COVID-19–related updates daily and were motivated to follow the news closely. However, as the pandemic persists, motivation to continue to learn about COVID-19 and risk-reducing actions may decrease [40], posing an additional challenge for policy makers who are trying to inform the public about updated safety measures. Further research should investigate the longitudinal patterns of public interest in health information to better tailor messages and choose information sources to control virus spread.

### Limitations

A limitation of the study was that for each individual participant, we treated trust in different sources independently; however, we acknowledge that participants tend to trust several sources rather than a single source exclusively. While elastic net regression accounted for relationships between sources, it would be interesting to explore if trust in different combinations of sources yields better knowledge and adherence. It is also important to note that we did not explore relationships between the frequency of news consumption, trust, and their joined association with knowledge. Focusing our questions on trust in specific sources allowed us to better understand whether participants take the information from a targeted source seriously. However, future research should explore in detail how the frequency of news consumption and trust of sources jointly influence participants’ knowledge and adherence. Finally, while we found significant results in the mediation analysis, the casual relationship should be interpreted in light of the fact that the data were collected in a cross-sectional survey [41,42] and that ultimately, the mediation model may have alternative causal explanations. For instance, compliance might be overreported by nonadherent participants who have accurate knowledge about what actions need to be taken (social desirability bias). Further longitudinal or experimental studies should replicate the mediation analysis reported in our work.

Lastly, while our sample demographics closely matched the White, African American, and Asian populations in the US, Hispanic respondents were underrepresented among our participants (18.3% as per US Census vs 7.5% in our data set) [25]. Future research should focus on a more detailed exploration of the associations between trust and knowledge in Hispanic populations.

### Conclusions

Distribution of accurate information through trusted sources is essential for facilitating public compliance with necessary health policies. Our work has identified a trend suggesting that trust in information sources varies among people of different ages and races. We recommend that policy makers use multiple sources to disseminate health information to ensure that different populations receive timely and accurate health information. Public trust in government-affiliated sources was positively associated with knowledge about COVID-19 and adherence to social distancing, whereas public trust in privately affiliated sources and social networks was negatively associated with knowledge and adherence. Private sources and social media must establish policies to control information quality to prevent the spread of misinformation, especially during a state of emergency, when inaccurate knowledge might contribute to public mortality.

## Appendix

Multimedia Appendix 1 Survey questions measuring knowledge about COVID-19 and social distancing behavior.

## Abbreviations

AUC: area under the curve
CDC: US Centers for Disease Control and Prevention
FDA: US Food and Drug Administration
OR: odds ratio
OSF: Open Science Framework
RMSE: root mean square error
WHO: World Health Organization
