# Supplementary material for: Association Between Public Knowledge About COVID-19, Trust in Information Sources, and Adherence to Social Distancing: Cross-Sectional Survey
Source: JMIR Public Health Surveill. 2020 Sep 15;6(3):e22060. doi: 10.2196/22060 (PMC7511226; doi:10.2196/22060)
Supplement: Multimedia Appendix 1 [file publichealth_v6i3e22060_app1.docx]

**Appendix**

**The Survey Items to Measure Knowledge:**

Instructions for participants: Please read the following statements and indicate whether you believe they are TRUE or FALSE.

- The United States is weeks away from having an FDA approved vaccine for coronavirus (F)
- Antibiotics can be used to treat the coronavirus (F)
- Most people who are infected with the coronavirus die from it (F)
- I cannot be infected if I wear a mask (F)
- People do not transmit the virus if they don’t have symptoms (F)
- Eating garlic can lower your chances of getting infected with the coronavirus (F)
- Most people who are infected with the coronavirus recover from it (T)
- By limiting the contact I have with people outside my household, I could prevent somebody's death (T)
- The main symptoms of the coronavirus are fever and cough (T)
- People of all ages can be infected with the coronavirus (T)
- People of all racial and ethnic groups can become infected with the coronavirus (T)
- To protect myself I need to wash hands frequently (T)

**The Survey Items to Measure Social Distancing:**

Instructions for participants: How often have you done the following in the past 7 days?

Response mode: 1 = Not at all, 2 = Once a week, 3= Several times a week, 4= Daily, 5=Several times a day

- Went to a gathering with 5 or more people.
- Hugged or touched someone who does not live with you
- Went inside someone else’s house.
- Had friends or family over to visit.
- Stood or walked close to someone who does not live with you
- Met face-to-face with people who don’t live with you

Instructions for participants: How often have you done the following in the past 7 days?

Response mode: 1=Always, 2=Usually, 3=Sometimes, 4=Rarely, 5= Never

- Stay 6 feet away from other people
